# Overexpression of SDF-1α Enhanced Migration and Engraftment of Cardiac Stem Cells and Reduced Infarcted Size via CXCR4/PI3K Pathway

**DOI:** 10.1371/journal.pone.0043922

**Published:** 2012-09-11

**Authors:** Kui Wang, Xiaohui Zhao, Chunyan Kuang, Dehui Qian, Hang Wang, Hong Jiang, Mengyang Deng, Lan Huang

**Affiliations:** Institute of Cardiovascular Diseases of PLA, Xinqiao Hospital, Third Military Medical University, Chongqing, People's Republic of China; Northwestern University, United States of America

## Abstract

Cardiac stem cells (CSCs) can home to the infarcted area and regenerate myocardium. Stromal cell-derived factor-1α/C-X-C chemokine receptor type 4 (SDF-1α/CXCR4) axis is pivotal in inducing CSCs migration. However, the mechanisms remain unclear. This study set out to detect if SDF-1α promotes migration and engraftment of CSCs through the CXCR4/PI3K (phosphatidylinositol 3-kinase) pathway. In the *in vitro* experiment, c-kit+ cells were isolated from neonatal mouse heart fragment culture by magnetic cell sorting. Fluorescence-activated cell sorting results demonstrated that a few c-kit+ cells expressed CD45 (4.54%) and Sca-1 (2.58%), the hematopoietic stem cell marker. Conditioned culture could induce c-kit+ cells multipotent differentiation, which was confirmed by cardiac troponin I (cTn-I), α-smooth muscle actin (α-SMA), and von Willebrand factor (vWF) staining. *In vitro* chemotaxis assays were performed using Transwell cell chambers to detect CSCs migration. The results showed that the cardiomyocytes infected with rAAV1-SDF-1α-eGFP significantly increased SDF-1α concentration, 5-fold more in supernatant than that in the control group, and subsequently attracted more CSCs migration. This effect was diminished by administration of AMD3100 (10 µg/ml, CXCR4 antagonist) or LY294002 (20 µmol/L, PI3K inhibitor). In myocardial infarction mice, overexpression of SDF-1α in the infarcted area by rAAV1-SDF-1α-eGFP infection resulted in more CSCs retention to the infarcted myocardium, a higher percentage of proliferation, and reduced infarcted area which was attenuated by AMD3100 or ly294002 pretreatment. These results indicated that overexpression of SDF-1α enhanced CSCs migration *in vitro* and engraftment of transplanted CSCs and reduced infarcted size via CXCR4/PI3K pathway.

## Introduction

Myocardial infarction (MI) is one of the leading causes of death and the ischemic injury is usually irreversible despite aggressive medical and revascularization treatment. Previous studies have shown that cardiac stem cells (CSCs) can differentiate into cardiomyocytes, smooth muscle cells or endothelial cells [1]–[4]. Therefore, transplantation of CSCs can reduce infarcted size and enhance heart function [1], [5]–[7], representing a new strategy for cardiac repair. At present, a variety of CSCs have been reported and classified according to biological markers such as c-kit+, side population and Islet-1+ [3], [8]. C-kit [9], also called KIT or CD117, is a cytokine receptor expressed on the surface of multiple stem cells. The c-kit+ CSCs represent one of the major CSCs populations which have properties of multidirectional differentiation [2], [3]. Animal and human studies [10]–[15] indicate that transplantation of c-kit+ CSCs reduces infarcted size and improves cardiac function of MI. However, the mechanism of c-kit+ CSCs homing to the infarcted zone is not clear.

Migration is a critical process for stem cells recruitment into target area and tissue repairing. Up to now, stromal cell-derived factor-1α (SDF-1α) and its cellular receptor CXCR4 (C-X-C chemokine receptor type 4) are known as the most prominent stem cell chemotaxis. SDF-1α has been shown to be significantly upregulated in many experimental models including myocardial infarction [5], [16] and attract the CXCR4+ stem cells towards SDF-1α gradient. Thus, upregulation of SDF-1α expression in ischemial myocardium represents a promising therapeutic strategy to improve post-infarction therapy. Recently, Tang et al [17] reported that exogenously expressed vascular endothelial growth factor (VEGF) promoted myocardial repair at least in part through SDF-1α/CXCR4-mediated migration of CSCs. However, the role of SDF-1α/CXCR4 pathway in CSCs migration needs to be further clarified.

Phosphatidylinositol 3-kinase (PI3K) is widely expressed and plays crucial roles in regulating multiple cell processes, such as cell migration, proliferation, differentiation, motility, survival and angiogenesis [18]–[20]. There are two distinct activities that PI3K participates in, phosphorylation of the regulatory subunit by the catalytic subunit and lipid phosphorylation. PI3K facilitates the transformation of phosphatidylinositol-3, 4-bisphosphate (PIP2) into phosphatidylinositol-3, 4, 5-trisphosphate (PIP3) which retrieves pleckstrin homology (PH) domain-containing proteins such as Akt to the cell membrane. Previous studies have demonstrated PI3K is also involved in SDF-1α/CXCR4-mediated chemotaxis and multiple stem/progenitor cells migration [18], [19], [21]. Overexpression of VEGF resulted in CSCs migration and homing to the injured myocardium, which was inhibited by the PI3K/Akt inhibitor [17].

The purpose of this study was to address the following questions: 1) whether overexpression of SDF-1α resulted in CSCs migration and accumulation in the infarcted region, and 2) whether SDF-1α-induced CSCs migration was mediated via CXCR4/PI3K signaling pathway.

## Materials and Methods

### Ethics statement

Animal experiments were approved by the Institutional Animal Care and Use Committee of Third Military Medical University. All the procedures were in compliance with the National Institute of Health Guide for the Care and Use of Laboratory Animals (NIH Publications No. 80-23).

### Isolation and culture of c-kit+ CSCs

C-kit+ cardiac stem cells were isolated from neonatal Bab/c mice (3–5 d) according to previous methods [2], [3], [22], [23]. Briefly, mouse hearts were removed and minced into small pieces, washed with phosphate buffered solution (PBS) and digested 3 times for 5 min at 37°C with a mixture of 0.2% trypsin (Invitrogen) and 0.1% collagenase IV (Sigma). Cells were filtered through 80-µm mesh and the remaining tissue fragments were washed with PBS and cultured as explants in complete explant medium (CEM) [IMDM(HyClone) supplemented with 10% fetal bovine serum (FBS) (Gibco), 100 Units/ml penicillin G, 100 µg/ml streptomycin, 2 mmol/L L-glutamine (Invitrogen) and 0.1 mmol/L 2-Mercaptoethanol (Sigma)] at 37°C and 5% CO_2_.

After 10 to 20 days, a layer of small, phasebright cells above the adherent explants were collected by washing with 0.5 g/L Trypsin-0.53 mmol/L EDTA. The cell suspension was filtered through a 40-µm cell strainer and isolated by magnetic cell sorting (MACS, Miltenyi Biotec) using anti-c-kit-coupled magnetic beads (Miltenyi Biotec).

The purified c-kit+ cells were seeded at 0.5–2×10^5^/ml in multiwell plates precoated with fibronectin in CGM(cardiosphere-growing medium) with minor modification [2], [23] [Dulbecco's MEM and Ham's F12 (ratio 1∶1, HyClone), 10% FBS, basic fibroblast growth factor (bFGF,10 ng/mL, Invitrogen), epidermal growth factor (EGF,20 ng/mL,Peperotech Inc), B27 (Invitrogen), thrombin (40 nmol/L,Sigma), LIF(1000 U/ml, Millipore), insulin–transferrin–selenite (Sigma), penicillin–streptomycin as in CEM ] at 37°C and 5% CO_2_.

### Flow cytometric analysis

Cells phenotypes were analyzed by fluorescence-activated cell sorting (FACS). For this purpose, purified c-kit+ cells were co-incubated for 30 min in the dark at room temperature with the following antibodies: phycoerythrin (PE)-conjugated antibodies against CXCR4, Sca-1 or CD45 (from BD Biosciences), and FITC-conjugated antibodies against c-kit. PE- or FITC-conjugated isotype IgG were used as control.10,000 to 50,000 cells were collected and subjected to flow cytometry using BD LSRII flow cytometer (BD Biosciences).

### Multiple differentiation of c-kit+ cells [23]

The purified c-kit+ CSCs were identified by immunofluorescence using a monoclonal antibody for c-kit. To induce *in vitro* differentiation [3], c-kit+ cells were seeded on 35-mm dishes(5×10^4^/dish) in DMEM [supplement with 10% FBS, 1 µM dexamethasone (Sigma), 50 µg/mL ascorbic acid (Sigma) and 1 mM β-glycerophosphate (Sigma)]. The medium was changed twice a week. Immunofluorescence analysis was performed with primary antibodies specific for cardiac troponin I (cTn-I) (1∶200, Santa Cruz), α smooth muscle actin (α-SMA) (1∶200, Zymed Lab) and von Willebrand factor (vWF) (1∶200, BD PharMingen). Goat anti-rabbit second antibody conjugated with FITC and TRITC were used for immunocytochemistry staining. Staining was observed by a Leica fluorescence microscopy (TCS SP5, Leica).

### rAAV1-SDF-1α-eGFP vector construction

Vector production, harvest, purification and testing were carried out as previously described [24]. The 267 bp mouse SDF-1α cDNA fragments combining with 3 bp stop codons were synthesized and amplified through polymerase chain reaction (PCR), inserted into the cloning sites between SalI and Bgl II in plasmid pSNAV2.0-mCMV-eGFP to construct the recombinant expression plasmid pSNAV2.0-SDF-1α-eGFP. The recombinant plasmid pSNAV2.0-SDF-1α-eGFP was co-transfected into HEK293 cells with the control plasmid pAAV-R2C1 and pHelper in AAV Helper-Free System for packaging of recombinant AAV. The efficiency of rAAV packaging was monitored under fluorescent microscope and recombinant viral particles were harvested from infected 293 cells. Viral titers were determined by dot blot analysis of DNA content and expressed as genome copies (gcs).

### Primary culture of neonatal mouse cardiomyocytes and rAAV infection

Neonatal mouse cardiomyocytes were isolated from 3–4 day Bab/c mice by adherence separation method [4]. Hearts were minced and digested at 37°C in 0.125% trypsinase and 0.08% collagenase II (sigma). Isolated cells were suspended in DMEM-L comprising 20% FBS, HEPES, L-glutamine and antibiotics. After 2 hours of adherence, cell suspensions were seeded in 24-well plates at a density of 1×10^5^ cells per well in a humidified condition. Two days later, the cardiomyocytes were infected with rAAV1-SDF-1α-eGFP at a MOI of 10^4^.

### Migration analysis

The migration of c-kit+ CSCs was assessed using 24-well transwell with an 8-µm pore size (Millipore) [25]. Cardiac myocytes were seeded on lower chamber and infected with rAAV1-SDF-1α-eGFP (infection group). Control group was infected with empty vector rAAV1-eGFP. Three days later, 5×10^4^ c-kit+ CSCs were added into the upper chamber. Migrated cells were stained with DAPI (Invitrogen) and counted in 10 high-power microscopic fields 24 hours later. For inhibition experiments, CSCs were pre-incubated with AMD3100 (10 µg/ml, Sigma–Aldrich) or LY294002 (20 µmol/L, Beyotime) for 1 h before seeding.

The SDF-1α protein concentration in lower chamber supernatant was determined by ELISA assay (R&D Systems) according to the manufacturer's instruction.

### Myocardial infarction model, CSCs and rAAV vectors injection

Mice were anesthetized by 1% pentobarbital (50 mg/kg, i.p.) and the left anterior descending coronary artery (LAD) was ligated by the 8-0 nonabsorbable surgical suture [26]. Myocardial infarction was verified by color change in the ischemic area.

The mice were then divided into 6 groups: 1. Sham group: the mice chest was opened but without LAD ligation. 2. MI group: ligation of LAD but without other treatment.3. Control (Empty) group: 50 µl empty vector and 1×10^5^ DAPI-labeled c-kit+ CSCs in 50 µl saline were injected into infarcted zone and infarcted border individually, with a 30 G needle at four points. 4. SDF-1α group: 50 µl rAAV1-SDF-1α-eGFP (2.25×10^12^ gcs/ml) and 1×10^5^ DAPI-labeled c-kit+ CSCs were injected in similar way. 5. AMD3100 group: 50 µl rAAV1-SDF-1α-eGFP injection and 1×10^5^ DAPI-labeled c-kit+ CSCs transplantation, however, pre-incubated with AMD3100 (10 µg/ml) for 1 hour. 6. LY294002 group: 50 µl rAAV1-SDF-1α-eGFP injection and 1×10^5^ DAPI-labeled c-kit+ CSCs transplantation, however, pre-incubation with LY294002 (20 µmol/L) for 1 hour.

The infection efficiency was evaluated by detection of GFP expression under confocal fluorescent microscope at day 21 and detection of SDF-1α expression by western blot respectively at day0, 4, 7, 14, and 21.

### Assessment of CSCs engraftment

To detect the engraftment of CSCs *in vivo*, 1×10^5^ DAPI-labeled c-kit+ CSCs in 50 µl saline were injected into infarcted border with a 30 G needle after coronary ligation. Three weeks after ligation, immunofluorence was performed in infarcted regions to detect the engraftment of DAPI-labeled cells. *In vivo* differentiation of CSCs was tested by immunohistochemistry using cTn-I antibody [27]. Ten confocal images were counted for engraftment and differentiation assay.

### Proliferation analysis of transplanted c-kit+ CSCs

Before being transplanted, c-kit+ CSCs were incubated with BrdU for 24 hours. Myocardial infarction model, rAAV-SDF-1α-eGFP injection and CSCs transplantation were operated as above. Three weeks later, BrdU was detected by immunofluorescent using a monoclonal antibody in border zones and infarcted region. The proliferation percentage of transplanted c-kit+ CSCs was caculated as the percentage of BrdU-positive nuclei vs total DAPI-stained nuclei.

### Infarcted size measurement

At day 21 post-MI, the infarcted size was measured by triphenyltetrazolium chloride (TTC) staining (n = 6) [28]. Briefly, the hearts were sliced transversely from the apex to base into 5 sections. Then, they were incubated for 30 min in 2% TTC (Sigma) solution at 37°C and fixed in 4% paraformaldehyde overnight. The infarcted myocardium (negative for TTC staining, white) and the non-ischemic zones (stained brick red) were divided and weighed. The infarction proportion was counted by ratio of infarcted mass/total heart.

### Western blotting

The expression of SDF-1α in infected zone was determined by western blot in 3 weeks. The mean densities of the bands were represented as the OD in units per square millimeter and normalized by GAPDH. The ratio of OD value of each group with control group served as the intensity of SDF-1α expression.

### Statistics

The data were analyzed using the SPSS 15.0 software. Continuous variables were expressed as mean ± SD. Multiple groups comparison was performed by one-way ANOVA followed by the Bonferroni procedure for comparison of means. A value of p<0.05 was considered significant.

## Results

### Culture of explants and c-kit+ CSCs

**Figure 1:**
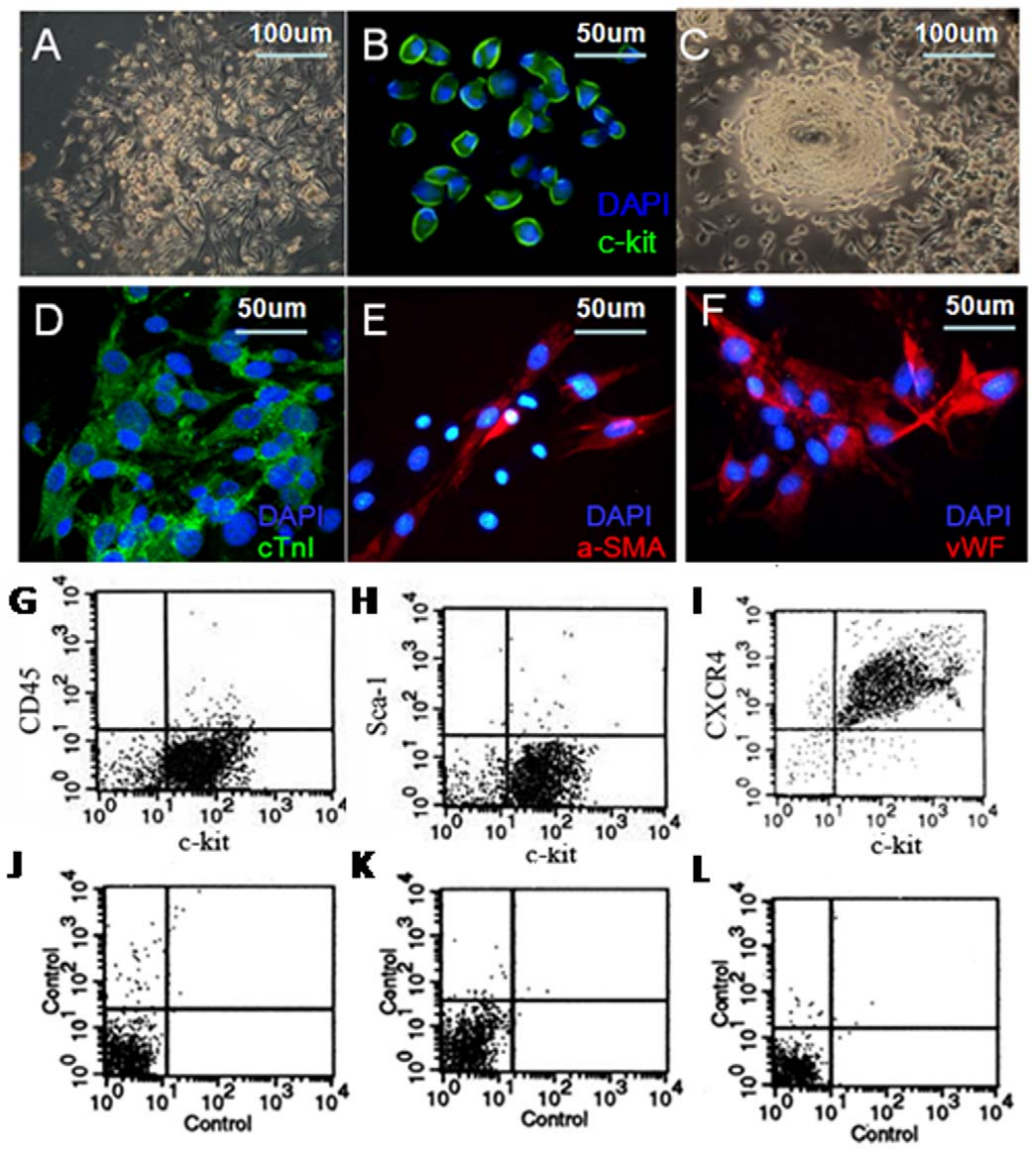
Culture and characterization of c-kit+ CSCs. **A**. small, round, phase-bright cells appeared. **B**. Purified c-kit+ cells were positive for c-kit staining (green). **C**. Phase-bright cells formed three-dimensional spheres. Multiple differentiation of c-kit+ cells into cardiomyocyte (**D**, green) smooth muscle (**E**, red), and endothelial cells (**F**, red). DAPI stained nuclei (blue). FACS analysis of c-kit+ cells phenotype with CD45 (4.54%±1.79%, G), Sca-1(2.58%±1.16%, H) and CXCR4 (91.47%±7.73%, I) (n = 3).

Five days after the heart tissues culture, fibroblasts migrated from the adherent explants. Then, some small, round and phase-bright cells appeared (Fig. 1A). After c-kit+ MACS isolation, these c-kit+ positive cells (green, Fig. 1B) were seeded in CGM and gradually formed three-dimensional spheres in 2 weeks (Fig. 1C).

### Multipotent differentiation of c-kit+ CSCs

C-kit+ cells changed their shape in conditioned culture. Confocal immunofluorescence analysis showed expression of cTn-I(green, Fig. 1D), cardiomyocyte specific protein, α-SMA (red, Fig. 1E), smooth muscle specific marker, and the endothelial specific marker-vWF(red, Fig. 1F).

### Flow cytometry analysis of cell surface markers

FACS of CD45, Sca-1 and CXCR4 co-stained with FITC-conjugated c-kit before MACS isolation were shown in Figure S1. However, 97% cells were c-kit positive after MACS isolation, and a few c-kit+ cells expressed CD45 (4.54±1.79%, Fig. 1G) and Sca-1 (2.58±1.16%, Fig. 1H), the marker of hematopoietic stem cells, indicating non-hematopoietic origin. Furthermore, we found high expression of CXCR4 (91.47±7.73%, Fig. 1I), the receptor of SDF-1α, in purified c-kit+ CSCs (n = 3). Controls with appropriate isotypes were shown in Fig. 1 J, K and L respectively.

### SDF-1α/CXCR4 induced CSCs migration via PI3K pathway

The determinations of recombinant plasmid pSNAV2.0-SDF-1α-eGFP and recombinated AAV1-SDF-1α-eGFP are shown in Supplementary Material S1. rAAV1-SDF-1α-eGFP construction was verified by PCR and immunofluorescence after infection (Figure S2). Three days after infection, over 90% cardiomyocytes expressed GFP and SDF-1α. (Figure S2).

**Figure 2:**
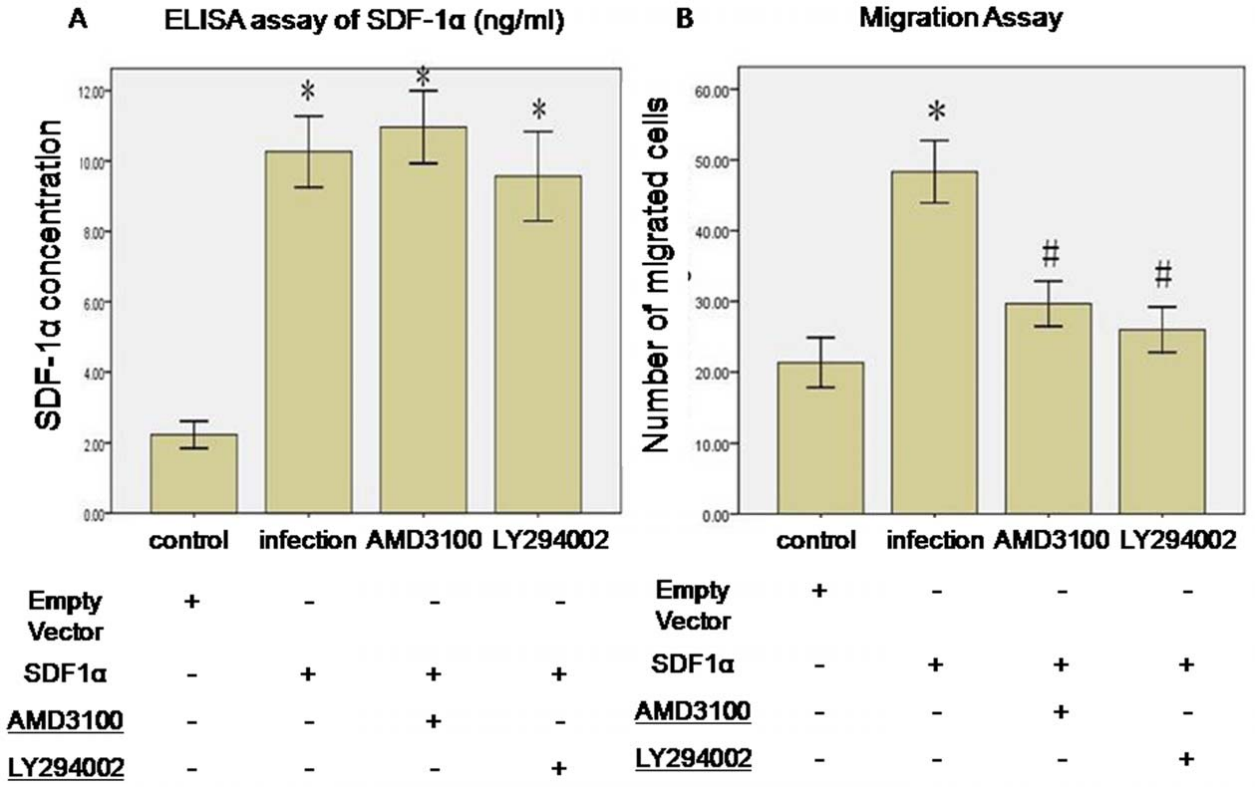
SDF-1α/CXCR4 induced CSCs migration via PI3K pathway. **A**. rAAV1-SDF-1α-eGFP infection increased SDF-1α concentration in supernatant. *p<0.01 vs. control. n = 3. **B**. SDF-1α-induced CSCs migration was inhibited by AMD3100 and LY294002. *p<0.01 vs. control. #p<0.05 vs. infection group. Data were shown as mean ± SD. n = 3.

ELISA assay indicated that significant higher SDF-1α concentration in infection group than control group (10.27±1.8 ng/ml vs. 2.23±0.67 ng/ml, p<0.01, n = 3). Also, there was no difference among SDF-1α, AMD3100 and LY294002 group (10.27±1.7 ng/ml vs. 10.97±1.8 ng/ml and 9.67±2.2 ng/ml, p>0.05, n = 3) (Fig. 2A). Overexpression of SDF-1α attracted more c-kit+ CSCs migration (infection vs. control group, 48.3±7.6 vs. 21.3±6.1, p<0.01). This effect was diminished by AMD3100 (29.7±6.0 vs. 48.3±7.6, p<0.05) and LY294002 (26.0±5.6 vs. 48.3±7.6, p<0.05) incubation (Fig. 2B).

### SDF-1α/CXCR4 induced CSCs engraftment via PI3K pathway

The efficiency of *in vivo* rAAV1-SDF-1α-eGFP infection is shown in Supplementary Material S1. Confocal fluorescence microscopy showed GFP expression in injected area (Figure S3A), indicating successful infection of rAAV1-eGFP and rAAV1-SDF-1α-eGFP. Also, SDF-1α expression peaked at day 14 and remained 2-fold elevated at day 21. (Figure S3B)

**Figure 3:**
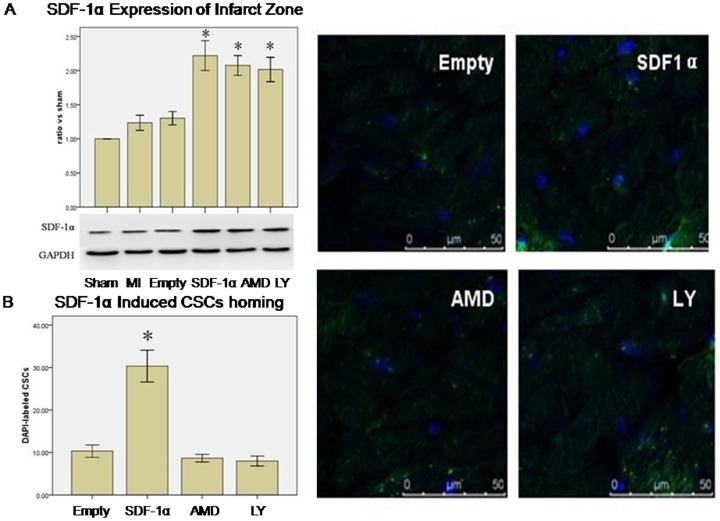
SDF-1α/CXCR4 induced CSCs homing via PI3K pathway. **A**. Western blotting analysis of SDF-1α expression in the infarcted zone 3 weeks after coronary artery ligation or sham operation (the ratio of SDF-1α expression vs. Sham group). * p<0.05 vs. Sham, MI and Empty (vector) group. GAPDH served as an internal control. Data were shown as mean ± SD. n = 3. **B**. SDF-1α Induced the CSCs Homing. Overexpression of SDF-1α induced DAPI-labeled CSCs homing to the infarcted zone. This effect was inhibited by AMD3100 and LY294002 pretreatment. * p<0.01vs. Empty, AMD(AMD3100) and LY(LY294002) group. Results were shown as mean ± SD, n = 6.

Western blot revealed that there was no difference of SDF-1α expression between MI and Empty group. However, infection with rAAV1-SDF-1α-eGFP significantly increased SDF-1α expression in infarcted myocardium (SDF-1α vs. MI, 2.22±0.38 vs. 1.20±0.19, p<0.01, SDF-1α vs. Empty, 2.22±0.38 vs. 1.30±0.17, p<0.01) at day 21. There was no difference among SDF-1α, AMD3100 and LY294002 groups (2.22±0.38 vs. 2.08±0.25 and 2.02±0.31, p>0.05, n = 3) (Fig. 3A).

Immunofluorence staining showed more CSCs in the infarcted regions in SDF-1α group (SDF-1α vs. Empty, 30.3±6.5 vs. 10.3±2.5, p<0.01 n = 6). However, the number of CSCs decreased in AMD3100 (AMD3100 vs. SDF-1α, 8.7±1.5 vs. 30.3±6.5, p<0.01) and LY294002 group (LY294002 vs. SDF-1α, 8.0±2.0 vs. 30.3±6.5, p<0.01) (Fig. 3B).

### Proliferation and differentiation of transplanted CSCs

**Figure 4:**
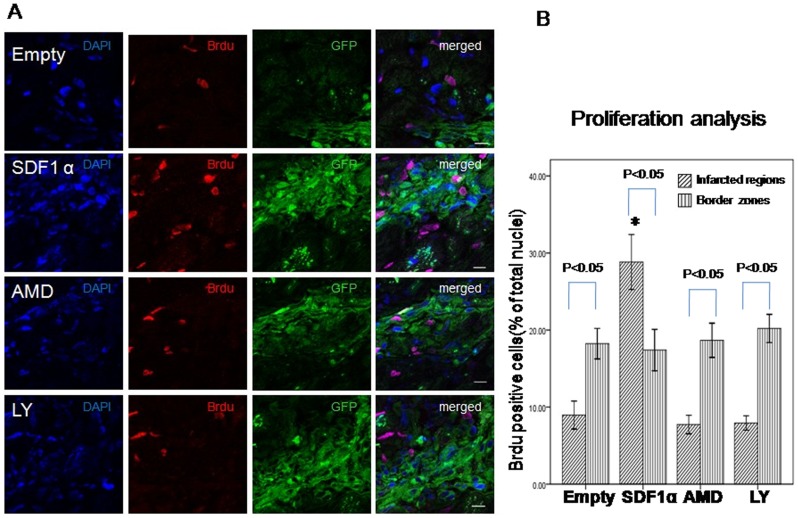
Proliferation of transplanted CSCs. **A**. proliferation of transplanted CSCs was determined by Brdu and DAPI double staining (red and blue) 3 weeks after myocardial infarction. Scale bar = 10 µm. **B**. Quantitative analysis of the ratio of Brdu/DAPI double positive cells. Data were shown as mean ± SD. * P<0.01 vs. Empty, AMD and LY group in infarcted zone. n = 6.

Proliferation of transplanted CSCs was determined by Brdu/DAPI double staining. Immunofluorence staining showed more BrdU positive cells in the infarcted regions in SDF-1α group than Empty group (SDF-1α vs. Empty, 28.8±1.5 vs. 8.9±1.8, p<0.01 n = 6) (Fig. 4). However, the cells number decreased in AMD3100 (7.7±1.7, p<0.01 vs. SDF-1α) and LY294002 group (7.9±0.9, p<0.01 vs. SDF-1α). Furthermore, more proliferated cells were detected in infarcted region in SDF-1α group than border zone (p<0.05) which contribute to the smaller infarcted area. There was no difference in border zone among all the groups.

**Figure 5:**
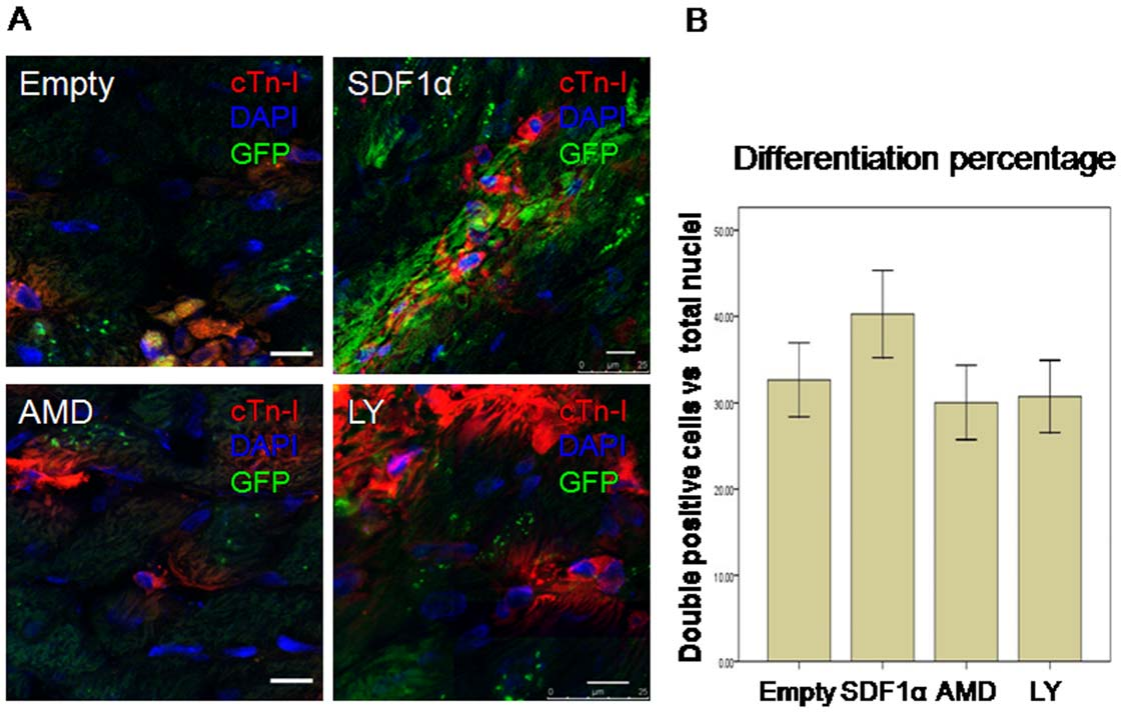
Differentiation of transplanted CSCs in infarcted zone. **A**. differentiation of transplanted CSCs was determined by cTn-I and DAPI double staining (red and blue) 3 weeks after myocardial infarction. Scale bar = 10 um. **B**. Quantitative analysis of the ratio of cTn-I/DAPI double positive cells. Data were shown as mean ± SD. n = 6.

To identify differentiation of CSCs, cTn-I staining was performed (Figure 5, red). Immunofluorence staining showed more DAPI-labeled cells around high density of green fluorescence which confirmed that overexpression of SDF-1α induced CSCs migration. Furthermore, we found that some DAPI-labeled CSCs were positive for cTn-I (red), indicating differentiation of homing CSCs into cardiomyocytes. Evidence for formation of novel myocytes was detected by the double positive of cTn-I and DAPI. However, we found that there was no difference of CSCs differentiation percentage among 4 groups in infarcted zone.

### SDF-1α/CXCR4 reduced MI size via PI3K pathway

**Figure 6:**
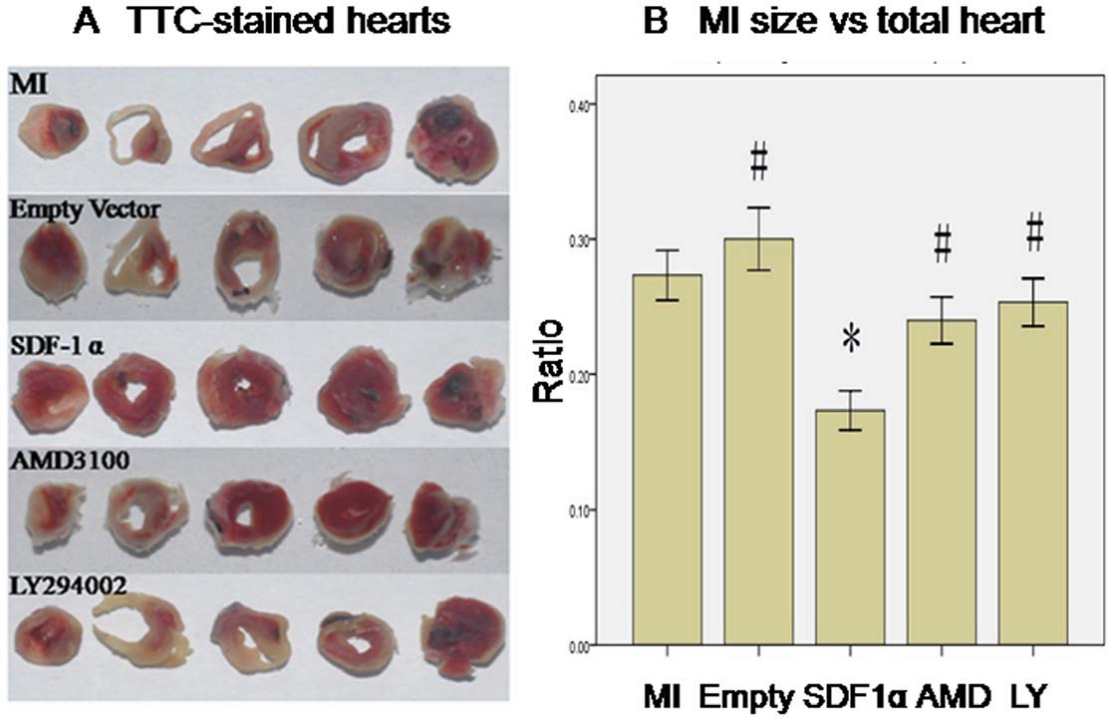
SDF-1α/CXCR4 reduced MI size via PI3K pathway. **A**. representative pictures of TTC-stained heart sections. (white, necrotic regions, not stained red by TTC). **B** graphic representation of the infarct size expressed as mass percentage of infarct size/total heart. *, P<0.05 vs. MI group, #, P<0.05 vs. SDF-1α group, n = 6.

TTC staining revealed empty vector infection didn't improve infarcted size (Empty vs. MI group, 30.0±4.0% vs. 27.3±3.2%, p>0.05, n = 6). However, rAAV-SDF-1α injection resulted in reduced infarcted size (SDF-1α vs. MI group, 17.3±2.5% vs. 27.3±3.2%, p<0.05, n = 6). This effect was abolished by AMD3100 pretreatment (SDF-1α vs. AMD3100 group, 17.3±2.5% vs. 24.0±3.0%, p<0.05, n = 6) and LY294002 pretreatment (SDF-1α vs. LY294002 group, 17.3±2.5% vs. 25.3±3.1%, p<0.05, n = 6) (Fig. 6).

## Discussion

Cardiac myocytes have been traditionally regarded as terminally differentiated cells that can be adapted to increased work and compensate for disease exclusively through hypertrophy [1]–[3]. In the past few years, compelling evidence proves that the heart has regenerative potential [1], [22]. Several types of CSCs have been recently identified according to the expression of some stem cell-related antigens including c-kit, MDR1, side population, Sca-1, Flk-1 or islet-1 [3], [9], [23], [29]. *In vitro* data have suggested that c-kit+ CSCs have a stronger growth potential than other types, although all of these CSCs categories can give rise to all cardiac cell lineages [9]. Recently, initial results of a randomized phase 1 trial [11] suggested intracoronary infusion of autologous c-kit+ CSCs was effective in improving LV systolic function and reducing infarcted size after myocardial infarction, thus, demonstrating that c-kit+ CSCs might be a perspective candidate for MI therapy.

Because there are no adequate resident CSCs to replace injured heart issue after MI [4], [10], [23], [29], [30], it is crucial to purify and expand CSCs *in vitro* for cardiac repair. We showed that c-kit+ cells kept their capacity for self-renewal and clonogenic *in vitro* with CGM medium, and could differentiate into cardiomyocytes, endothelial cells and smooth muscle cells. FACS analysis showed that they were not hematopoietic stem cells.

SDF-1α is a member of the CXC or a chemokine subfamily and the only known ligand for the chemokine receptor CXCR4 [19]. SDF-1α is constitutively expressed in a large number of tissues and plays an important role in multiple stem cells migration by binding CXCR4 [5], [16], [17], [19], [25]. Yu et al reported that MI in rat hearts led to an increased expression of SDF-1α, which mediated migration of BMSCs through SDF-1α/CXCR4 and activation of PI3K/Akt [18]. However, the role of SDF-1α/CXCR4 pathway in CSCs migration is still needed to be further clarified. a *et al* reported SDF-1α expression was significantly higher within 4 days post operation in the MI groups and peaked at 1 day post MI [31]. In our present study, we did not observed a high expression of SDF-1α in the Empty and MI groups at day 21 after MI which might be due to the death of infarcted myocardium. Also, previous study found that myocardial infarction increased expression of SDF-1α mRNA by 56.7% and 95.7% at 48 and 72 hours, respectively, and returned to baseline by 7 days. SDF-1α protein level peaked at 72 hours and remained 2-fold elevated at 96 hours [32]. Thus, the SDF-1α expression in infarcted zone decreased rapidly and it is reasonable that endogenous SDF-1α is not sufficient for CSCs engraftment and myocardial repair in chronic phase-a suitable time for stem cells survival after acute inflammatory response. Recently, Tang et al [33] reported that exogenously expressed VEGF promoted myocardial repair at least in part through SDF-1a/CXCR4-mediated recruitment of CSCs. Considering of rare CSCs in the heart, we focused on the effect of CSCs transplantation in combination with exogenously overexpression of SDF-1α in chronic phase after MI, which might be better associated with clinical feasibility.

Our result suggests that SDF-1α overexpression *in vivo* could promote the engraftment of the locally transplanted CSCs into the infarcted region and cardiac repair. Blocking CXCR4 with AMD3100 can eliminate these effects, indicating an essential role of SDF-1α/CXCR4 axis in CSCs migration and recruitment. CXCR4 is a Gi-coupled receptor. Studies have shown that SDF-1α, after binding to CXCR4, causes mobilization of calcium, decreases of cyclic AMP within the cells, and activates multiple signaling pathways such as PI3K [34]. The activation of the PI3K-Akt pathway can occur as a result of G-protein-coupled receptor triggering- through direct interaction with the active G protein ^β^γ subunit, or indirectly, by α subunit-induced tyrosine kinase activity [35]. PI3K catalyzes the phosphorylation of membrane phosphatidylinositols generating phosphatidylinositol mono-, bis-, and tris-phosphate [36]. These products recruit the protein kinase Akt by interacting with its PH domain and facilitate its phosphorylation on threonine 308 and serine 473 by phosphoinositide-dependent kinase 1 and 2 [37]. Ultimately, SDF-1α-bound CXCR4 induces cytoskeletal rearrangement, adhesion to endothelial cells, polarized migration of cells to specific organs and the secretion of angiopoietic factors, all important components of the repair process [38], [39].

Although previous experiment has provided direct evidence that the PI3K pathway is required for SDF-1α-induced cell migration in HPC, BMSC [16], [18] and VEGF-induced CSCs migration [17], there are no related report involving SDF-1α action on CSCs. We found that administration of the specific PI3K inhibitor LY294002 significantly inhibited the SDF-1α-induced CSCs migration and engraftment, indicating the important role of PI3K pathway. Furthrmore, as a known downstream effector of the PI3K dependent signaling cascade, Akt is required for SDF-1α-mediated chemotaxis, cell proliferation and regulation of intergrin [18], [19], [40] and thus may play a role in SDF-1α/CXCR4 mediated CSCs migration and recruitment.

In addition to CSCs, stem cells and/or progenitors from the bone marrow, such as mesenchymal stem cells (MSCs) [18], hematopoietic stem cells (HSCs), and endothelial progenitor cells (EPCs) [16], [19], may be recruited to participate in the repair process of ischemic myocardium through SDF-1α/CXCR4 axis. Thus, the beneficial effects of SDF-1α overexpression on myogenesis and angiogenesis in the ischemic heart, as demonstrated in our experiments, may also involve these cells from extracardiac sources.

We also detected the fate of transplanted CSCs 3 weeks after MI. Although there was no difference of differentiated CSCs among 4 groups in infarcted area, more proliferated CSCs in infarcted zone in SDF-1α group were disclosed than other groups. It could be the reason of improved infarcted size after SDF-1α overexpression.

In conclusion, the present study showed that overexpression of SDF-1α induced CSCs migration *in vitro* and engraftment of transplanted CSCs and reduced infarcted size via CXCR4/PI3K pathway which representing a novel therapeutic strategy for MI.

## Supporting Information

Figure S1 **FACS of cultured cells before c-kit MACS isolation.**
(TIF)

Figure S2 **Determination of rAAV1-SDF-1α-eGFP.**
(TIF)

Figure S3 **The efficiency of** ***in vivo*** **rAAV1-SDF-1α-eGFP infection.**
(TIF)

Supplementary Material S1 **Supplementary methods and results.**
(DOC)
