# Supplementary figures and images for: Overexpression of SDF-1α Enhanced Migration and Engraftment of Cardiac Stem Cells and Reduced Infarcted Size via CXCR4/PI3K Pathway
Source: PLoS One. 2012 Sep 11;7(9):e43922. doi: 10.1371/journal.pone.0043922 (PMC3439464; doi:10.1371/journal.pone.0043922)

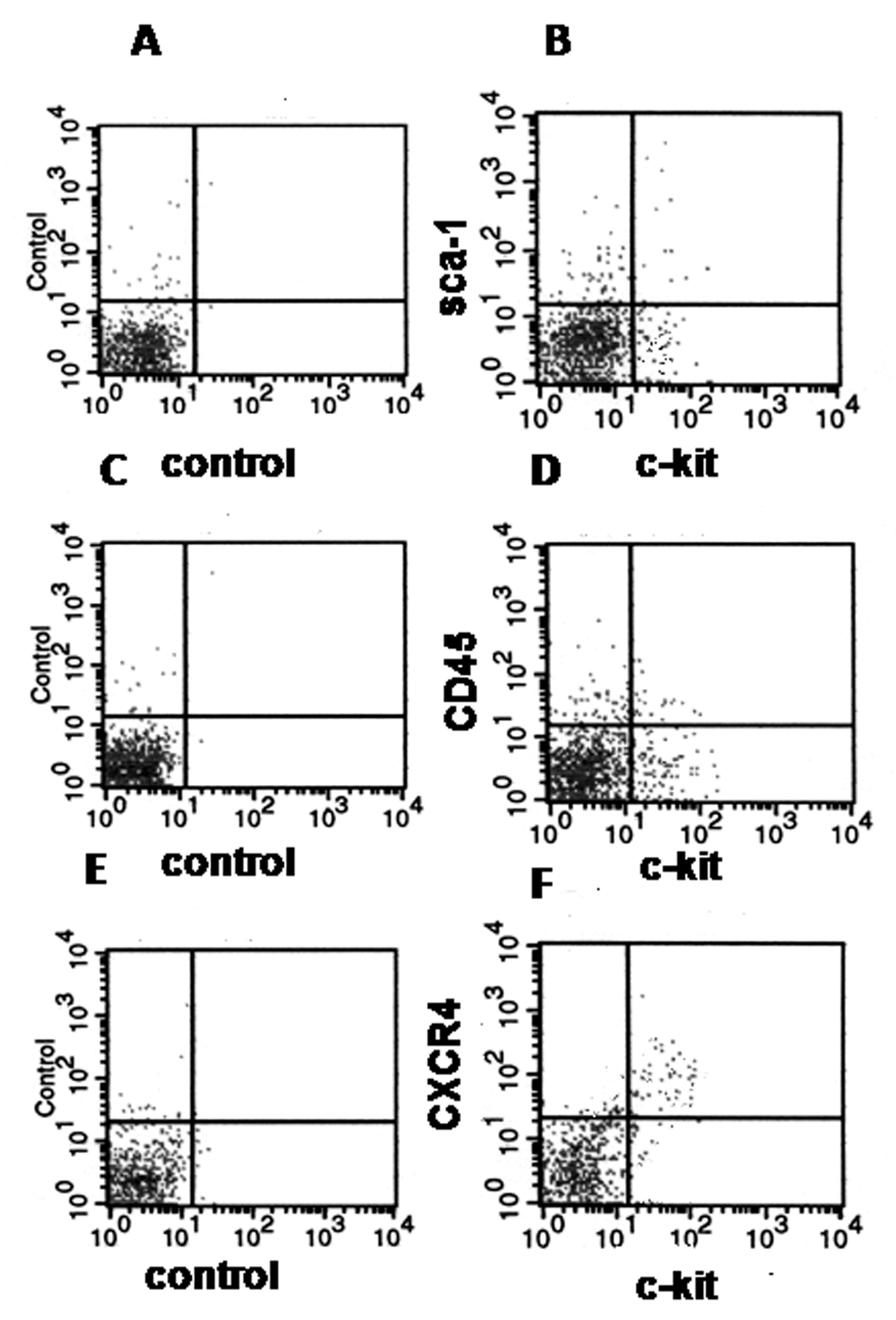

Supplement: Figure S1 — FACS of cultured cells before c-kit MACS isolation. (TIF) [file pone.0043922.s001.tif]

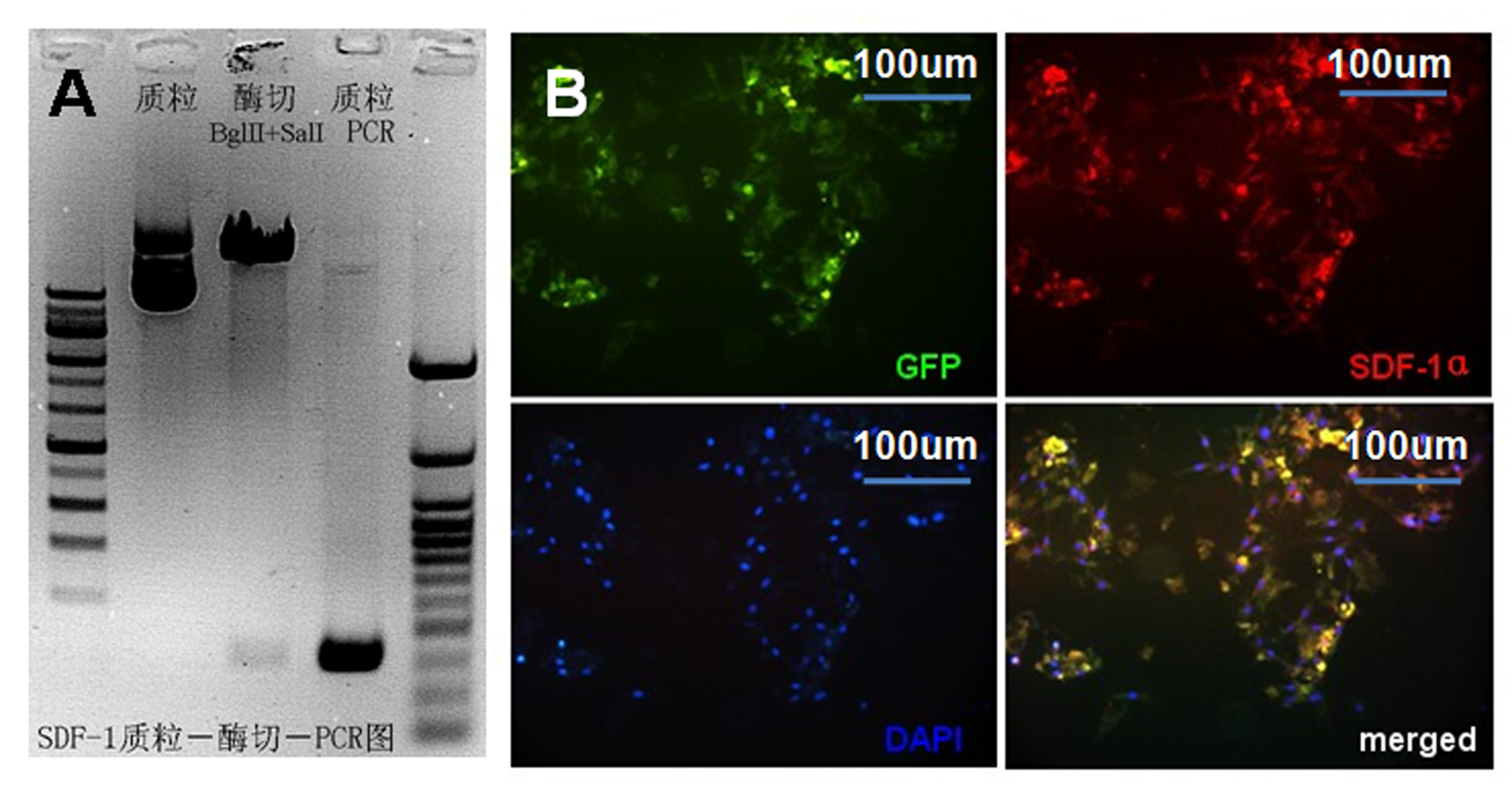

Supplement: Figure S2 — Determination of rAAV1-SDF-1α-eGFP. (TIF) [file pone.0043922.s002.tif]

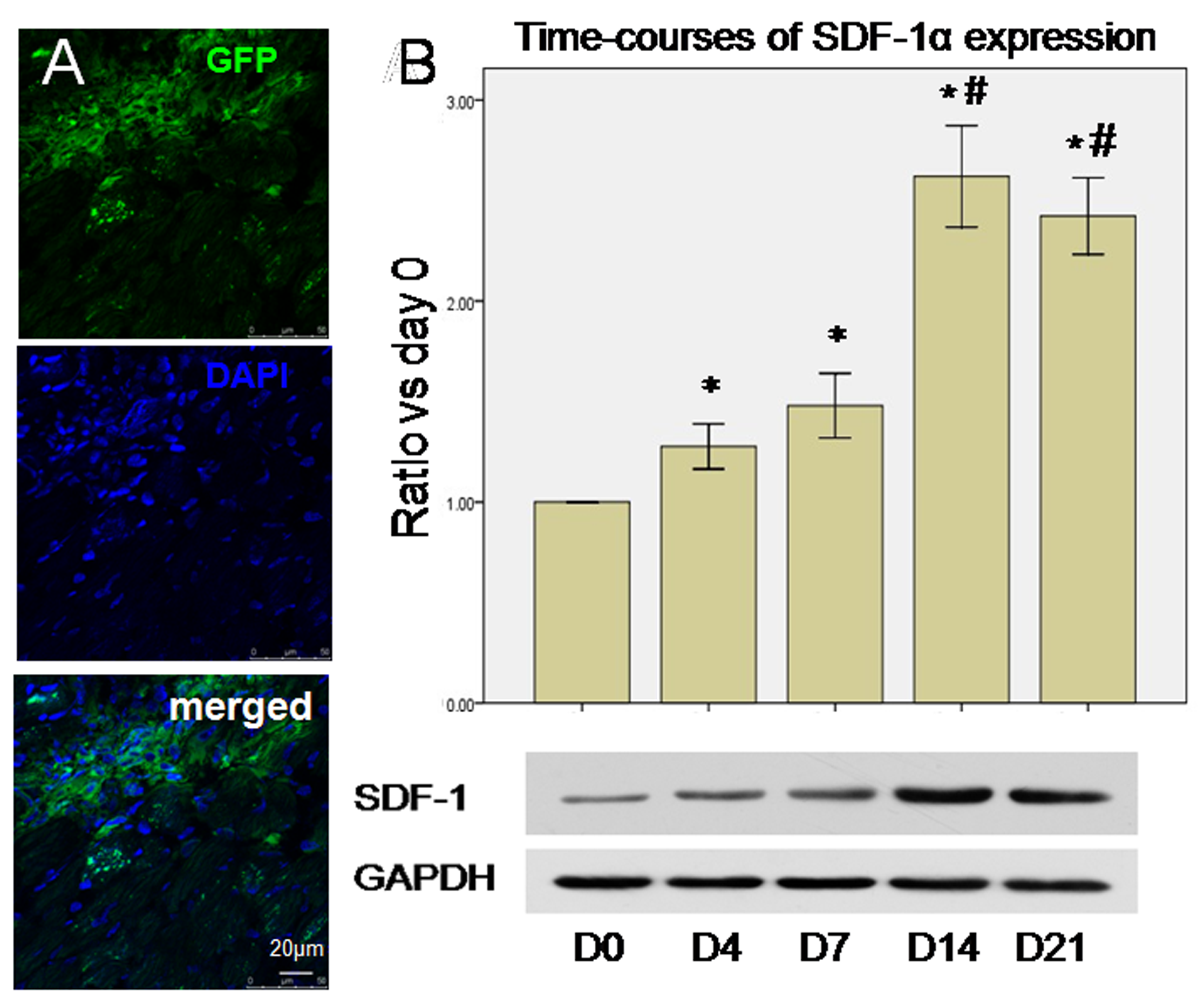

Supplement: Figure S3 — The efficiency of in vivo rAAV1-SDF-1α-eGFP infection. (TIF) [file pone.0043922.s003.tif]
