# Supplementary material for: Overexpression of SDF-1α Enhanced Migration and Engraftment of Cardiac Stem Cells and Reduced Infarcted Size via CXCR4/PI3K Pathway
Source: PLoS One. 2012 Sep 11;7(9):e43922. doi: 10.1371/journal.pone.0043922 (PMC3439464; doi:10.1371/journal.pone.0043922)
Supplement: Supplementary Material S1 — Supplementary methods and results. (DOC) [file pone.0043922.s004.doc]

**Supplementary materials**

Supple 1. Characterization of cell surface markers before MACS (Figure S1).

**Figure S1.** FACS of cultured cells before c-kit MACS isolation. c-kit+ cells accounted for 8.3% of the total unsorted cells. A few cells were double positive for c-kit/sca-1(1.05%±0.16%, Fig.1B), c-kit/CD45 (0.87%±0.12%, Fig.1D) and c-kit/CXCR4 (5.62%±0.54%, Fig.1F). Controls with appropriate isotypes were shown in left lane figure 1. A, C and E respectively. (n=3).

Supple 2

Recombinant plasmid pSNAV2.0-SDF-1α-eGFP was determinated by PCR, restriction enzyme digestion and gene sequencing. The recombinated AAV1-SDF-1α-eGFP was determinated by observing GFP expression and detecting SDF-1α in infected cardiomyocytes. The PCR result showed PCR could amplify an about 270 bps band, consistent with the cDNA length of SDF-1α ([Fig](http://pt.wkhealth.com/pt/re/circres/fulltext.00003012-200703020-00019.htm;jsessionid=HXvTTRlF8JvCR8XlvJrryj1q10sVBJpZTZKyzz4G7H58w21hKJP7!65375592!181195628!8091!-1#FF1%23FF1)ure S2A). Gene sequencing showed that the inserted sequence was fully consistent with the SDF-1α cDNA (not shown). In cardiomyocytes infected with rAAV1-SDF-1α-eGFP, almost all cells expressed GFP ([Fig](http://pt.wkhealth.com/pt/re/circres/fulltext.00003012-200703020-00019.htm;jsessionid=HXvTTRlF8JvCR8XlvJrryj1q10sVBJpZTZKyzz4G7H58w21hKJP7!65375592!181195628!8091!-1#FF1%23FF1)ure S2B, green). Furthermore, SDF-1α could be detected in infected cardiomycytes by an anti-SDF-1α antibody and TRITC-conjugated second antibody under fluorescent microscope (Figure S2B, red). These results manifested that rAAV construction was successful and infection was effective.

**Figure S2.** Determination of rAAV1-SDF-1α-eGFP. **A**. PCR and restriction enzyme digestion generated a 270 bps band, consistent with the cDNA length of SDF-1α. **B**. In infected cardiomyocytes, almost cells expressed GFP and SDF-1α, indicating successful infection.

Supple 3

The efficiency of *in vivo* infection was evaluated by detection of GFP expression in myocardium under confocal fluorescent microscope after 3 weeks of rAAV1-SDF-1α-eGFP injection. The time-course of the infection efficiency was assessed by SDF-1α protein expression respectively at day 0,4,7,14,21. The SDF-1α expression was normalized by GAPDH. The ratio of OD value ​​of each group with the day 0 group served as the intensity of SDF-1α expression

GFP expression was observed in infarcted region (Figure S3A). SDF-1α expression significantly increased at day 4(1.28±0.11, p<0.05), peaked at day 14 (2.62±0.25, p<0.01 vs. day 0) and remained high expression at day 21(2.42±0.19, p<0.01 vs. day 0). (Figure S3B, n=3).

**Figure S3.** The efficiency of *in vivo* rAAV1-SDF-1α-eGFP infection. **A**. GFP expression in infarcted regions. **B**. The time-course of SDF-1α expression in infarcted myocardium after rAAV1-SDF-1α-eGFP infection. SDF-1α expression significantly increased at day 4(1.28±0.11, p<0.05 vs. day 0), peaked at day 14 (2.62±0.25, p<0.01 vs. day 0) and remained high expression at day 21(2.42±0.19, p<0.01 vs. day 0). (n=3)
